# Multiplex PCR-Based Neutralization (MPBN) Assay for Titers Determination of the Three Types of Anti-Poliovirus Neutralizing-Antibodies

**DOI:** 10.3390/vaccines8010120

**Published:** 2020-03-05

**Authors:** Hasmik Manukyan, Svetlana Petrovskaya, Konstantin Chumakov, Majid Laassri

**Affiliations:** Division of Viral Products, Center for Biologics Evaluation and Research, US Food and Drug Administration, 10903 New Hampshire Avenue, Silver Spring, MD 20993, USA; hasmik.manukyan@fda.hhs.gov (H.M.); svetlana.petrovskaya@fda.hhs.gov (S.P.); konstantin.chumakov@fda.hhs.gov (K.C.)

**Keywords:** neutralization, anti-poliovirus antibodies seroprevalence, OPV, IPV, clinical trials, immunogenicity

## Abstract

Determination of poliovirus-neutralizing antibodies is an important part of clinical studies of poliovirus vaccines, epidemiological surveillance and seroprevalence studies that are crucial for global polio eradication campaigns. The conventional neutralization test is based on inhibition of cytopathic effect caused by poliovirus by serial dilutions of test serum. It is laborious, time-consuming and not suitable for large scale analysis. To overcome these limitations, a multiplex PCR-based neutralization (MPBN) assay was developed to measure the neutralizing antibody titers of anti-poliovirus sera against three serotypes of the virus in the same reaction and in shorter time. All three anti-poliovirus sera types were analyzed in a single assay. The MPBN assay was reproducible, robust and sensitive. Its lower limits of titration for the three anti-poliovirus sera types were within range of 0.76–1.64 per mL. Different anti-poliovirus sera were tested with conventional and MPBN assays; the results obtained by both methods correlated well and generated similar results. The MPBN is the first neutralization assay that specifically titrates anti-poliovirus antibodies against the three serotypes of the virus in the same reaction; it can be completed in two to three days instead of ten days for the conventional assay and can be automated for high-throughput implementation.

## 1. Introduction

Poliomyelitis is an infectious viral disease caused by the three serotypes of poliovirus. It affects the central nervous system and can cause temporary or permanent paralysis. There are two excellent vaccines that protect against poliomyelitis. Inactivated poliovirus vaccine (IPV) was developed by Jonas Salk in 1955 [1] and is administered by injection. The live attenuated poliovirus vaccine (OPV) developed by Albert Sabin in 1961 is administered orally [2,3]. Both vaccines are highly effective; however, recipients of OPV excrete mutant variants of Sabin strains [4,5] in stools that can become pathogenic vaccine-derived polioviruses and circulate in the population, occasionally causing disease in unvaccinated persons [6,7]. The limitation of IPV is that it elicits poor intestinal immunity and is not able to stop virus circulation [8,9]. Therefore, new improved poliovirus vaccines are under development [10,11,12,13]. The absence of significant poliovirus morbidity makes clinical evaluation of the new vaccines based on protection endpoint impossible. Therefore, their efficacy can only be assessed using immunogenicity endpoint. Serum neutralizing antibodies present at 1:8 or higher level represent a well-established biomarker of protection. In addition, the serum neutralization test is a crucial part of the rat potency test for IPV. Poliovirus neutralizing antibody titer is determined by a standard poliovirus neutralization test [14,15] using serial dilutions of serum samples that are incubated in 96-well microtiter plates with a fixed amount of poliovirus. After the incubation, a suspension of susceptible cells is added to the serum-poliovirus complex and incubated for 10 days [14]. The standard poliovirus neutralization test requires personnel expertise to observe and interpret the cytopathic effect (CPE) in infected cells, takes 7 to 10 days to generate the results and is not suitable for large-scale analysis. Determination of neutralizing antibodies to each of the three serotypes of poliovirus must also be performed in a separate assay. Critically, the test produces variable results [16,17].

In the end-game of the polio eradication program, surveillance of seroprevalence against poliovirus in susceptible populations is fundamental for monitoring the exposure to poliovirus circulation in poliovirus-free countries to maintain the poliovirus-free status and the seroconversion rates in countries where poliovirus is endemic to assess the efficacy of vaccination programs. In these cases, it is important to generate data about the presence of anti-poliovirus antibodies in the shortest time possible and high-throughput manner to expedite the development of the improved poliovirus vaccines and to analyze the seroprevalence of population needed for the poliovirus eradication end-game. Therefore, a faster multiplex and high-throughput assay would be a desirable alternative to the conventional assay.

This communication describes the development of a multiplex PCR-based neutralization (MPBN) assay for anti-poliovirus antibodies titration that uses a quantitative multiplex one-step RT-PCR (qmosRT-PCR) [18] as a read-out instead of CPE used in the conventional assay. Recently, we successfully used a similar approach to develop a multiplex PCR-based titration (MPBT) assay for the simultaneous titration of the three poliovirus serotypes [19].

The MPBN assay generates similar results as the conventional neutralization assay while reducing the time to generate results and the variation that is due to the interpretation of CPE. It is reproducible, sensitive, robust and allows titration of anti-poliovirus sera against all three serotypes in the same reaction.

## 2. Materials and Methods

### 2.1. Hep-2C Cells, Viruses and Sera

HEp-2C cells (ATCC^®^ CCL-23^TM^), derived from a human carcinoma, were cultured in 175-cm^2^ flasks at 37 °C ± 2 °C in Dulbecco’s modified Eagle’s medium (DMEM; ThermoFischer, Frederick, MD, USA) supplemented with 5% fetal calf serum (FCS; ThermoFischer, Frederick, MD, USA) and penicillin-streptomycin (100 U/mL and 100 µg/ mL, respectively; ThermoFischer, Frederick, MD, USA). Viable cells were sampled, stained with Trypan Blue (ThermoFischer, Frederick, MD, USA) and counted with a hemocytometer (Countess^TM^; ThermoFischer, Frederick, MD, USA). One hundred microliters of cell suspension containing 4 × 10^4^ cells for MPBN assays and 1–2 × 10^4^ cells for conventional neutralization assay was added to each well of a 96-well microplate.

Lots of US neurovirulence poliovirus reference vaccines (Sabin type 1, 2 and 3 OPV strains having GenBank accession numbers AY184219, AY184220 and AY184221, respectively) were used as challenges to compare results of the conventional neutralization and MPBN assays. Poliovirus sera were obtained from our Laboratory of Method Development stock via Dr. Diana Kouiavskaia (CBER, FDA).

### 2.2. Quantitative Multiplex One-Step RT-PCR

The quantitative multiplex one-step RT-PCR (qmosRT-PCR) was described previously [18]. Briefly, the qmosRT-PCR reactions were prepared in 96-well optical plates in a final volume of 25 μL using 2 μL of 1/20 diluted cell lysate and QuantiFast Multiplex RT-PCR Kit (QIAGEN, Valencia, CA, USA). The diluted lysates of cells infected with Sabin 1, 2 and 3 were used as positive control, and water and diluted lysates of noninfected cells were used as negative controls. All control samples were run in duplicates. The specific primer pairs and probes used for each Sabin strain were: for Sabin 1, forward primer—2771Sab1F, 5′CAGCTTCCACCAAGAATAA3′, reverse primer—3036Sab1R, 5′GATTGATGGATTTGATGAG3′ and probe—Sab1-FAM, FAM-5′ACAGTGTGGAAGATC3′-NFQ; for Sabin 2, forward primer—2682TqS2F, 5′CCAGAGACGAACGCGA3′, reverse primer—2803TqS2R, 5′CAAACCGAAAACAATCTGC3′ and probe—Sab2-VIC, VIC-5′CACGGTTGAGTCATTC3′-NFQ, and, for Sabin 3, forward primer—1411TqS3F 5′GGGAAAATTTTACTCCCAA3′, reverse primer—1629TqS3R 5′TGAATCAATGGCCAAAGCA3′ and probe—Sab3-NED, NED-5′AACGCAGTAACATCC3′-NFQ.

The primers were used at a concentration of 0.4 μM for each primer of poliovirus type 2 and 0.8 μM for each primer of poliovirus type 1 and 3. The TaqMan probes were used at concentration 25 nM for each probe of each poliovirus serotype. The qmosRT-PCR procedure was performed using real-time PCR System ViiA7 (Applied Biosystems, Foster City, CA, USA) at the following thermal cycling conditions: one cycle incubation for 20 min at 50 °C and 5 min at 95 °C, followed by 45 cycles, each consisting of 15 s at 95 °C, 15 s at 50 °C and 50 s at 60 °C.

### 2.3. Conventional Neutralization Assay

The conventional neutralization assay was performed using Sabin 1, 2 and 3 strains and a HEp-2C cell line according to the World Health Organization (WHO) manual [15]. Briefly, the titers of neutralizing antibodies against poliovirus serotypes were determined using 96-well microplates. The sera were inactivated at 56 °C for 30 min and subsequently diluted from 1:8 to 1:2048 in two-fold serial dilutions; this dilution was performed in 25 μL. To each well, 25 μL of poliovirus (100 CCID_50_/25 μL) was added and incubated for 3 h at 36 °C to allow the antibodies to bind to poliovirus. Next, one-hundred-µL aliquots of cell suspension containing 1–2 × 10^4^ HEp-2C cells in DMEM with 2% FCS were added to each well of diluted sera-virus complex in triplicate wells of 96-well plates for each sample. Virus-infected plates were incubated for ten days at 36 °C in a 5% CO_2_ humid atmosphere; wells were periodically inspected for the presence of cytopathic effect (CPE). Wells showing CPE were counted on day ten after infection and sera titers calculated using the Spearman-Karber formula [20].

### 2.4. MPBN Assay and Data Analysis

Like the conventional neutralization assay, the MPBN assay was performed using Sabin 1, 2 and 3 strains and a HEp-2C cell line according to the WHO manual [15]. The titers of neutralizing antibodies against poliovirus serotypes were determined using the 96-well microplates. The sera were inactivated at 56 °C for 30 min and subsequently diluted from 1:8 to 1:2048 in two-fold serial dilutions; this dilution was performed in 25 μL per well of a 96-well plate. To each well, 25 μL of the three Sabin strains (100 CCID_50_ of each) was added and incubated for 3 h at 36 °C to allow the antibodies to bind to poliovirus. Next, one-hundred-µL aliquots of cell suspension containing 4 × 10^4^ HEp-2C cells in DMEM with 2% FCS were added to each well of viruses-sera mixture. The plates were then incubated at 36 °C in a 5% CO_2_ humid atmosphere for 42 h. Next, the viral medium was aspirated (this allows us to detect the replication of only those viruses that enter the cell) from the wells, and 50 μL of 0.9% Triton X-100 in DMEM was added to each well. The plates were sealed with a foil and stored at −80 °C prior to qmosRT-PCR analysis.

The plates with the triton-lysed cells were thawed for 30 min at room temperature and briefly centrifuged in a 5810R centrifuge (Eppendorf, San Diego, CA, USA) at 1000 rpm for 1 min to collect the liquid at the bottom of the wells. The 1:20 dilutions were prepared by adding 10 μL of the cell lysates to 190 μL of molecular biology grade water (5-PRIME, Gaithersburg, MD, USA).

The three poliovirus serotypes were quantified in the same reaction of qmosRT-PCR as described previously [18] and summarized above. The positive and negative results for viral replication were recorded for each well and used for sera titers calculation for each poliovirus serotype in accordance with the Spearman-Karber formula [20].

The samples that had Ct less or equal than 40 were considered positive, and samples with Ct higher than 40 were considered negative. The results of the PCR were considered nonvalid if both reactions of the positive control gave negatives results and/or both reactions of the negative controls gave positive results.

## 3. Results

### 3.1. MPBN Assay Design

To test whether all three Sabin strains can be identified and quantified simultaneously in one multiplex reaction, lysates of HEp-2C cells infected with Sabin strains were prepared in different combinations and analyzed by quantitative multiplex one-step reverse-transcriptase PCR (qmosRT-PCR) [18]. The results summarized in Table 1 show that qmosRT-PCR was able to specifically identify and quantify each Sabin strain in all possible combinations. Consistent with our previous studies [18,19,21], qmosRT-PCR was found to be very specific for the identification and quantification of the different Sabin strains.

**Figure 1:**
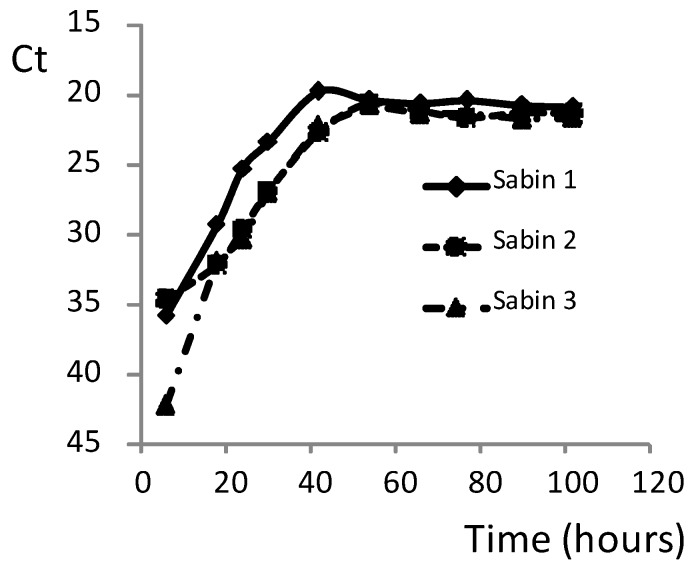
Dynamic growth of the three Sabin strains in HEp-2C cells infected with 100 CCID50 of each of the three Sabin strains simultaneously. Ct: quantitative PCR cycle threshold.

To study the growth kinetics of Sabin strains in cells infected with the same virus concentration as used in the neutralization tests, monolayers of HEp-2C cells were prepared in 96-well plates and infected with 100 CCID_50_/per well of a mixture of the three Sabin strains. The cells were lysed at different time-points and analyzed with qmosRT-PCR, as described above. The resulting Ct values were plotted against harvest times (Figure 1). The result showed that all Sabin strains reached maximum growth at 36–42 h post-infection. Therefore, this time was chosen as the optimum time-point for cells lysis before applying qmosRT-PCR.

**Figure 2:**
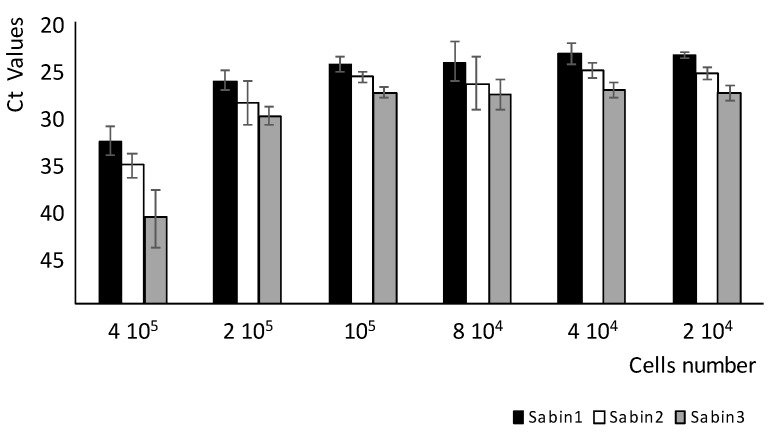
Effect of cell numbers on Sabin strains quantitation.

In the next experiments, the effect of the number of cells in the assay on virus quantitation was studied. Different number of HEp-2C cells were added to the mixture of 100 CCID_50_ of each of the three Sabin strains and incubated for 42 h. Next, the cells were lysed and analyzed with qmosRT-PCR as described above. The resulted Ct values were plotted against the number HEp-2C cells (Figure 2). This result showed that 2 × 10^4^ to 2 × 10^5^ cells harvested at 42 h resulted in consistent and the lowest Ct values (i.e., highest number of viral genome copies) for all three Sabin strains. Therefore, the cells number of 4 × 10^4^ cells was chosen as an optimum number of cells for the assay. This wide interval of cell number (2 × 10^4^ to 2 × 10^5^cells) that resulted in the highest number of the quantified viral genome copies suggests the robustness of the MPBN assay (see Section 3.3 below).

**Figure 3:**
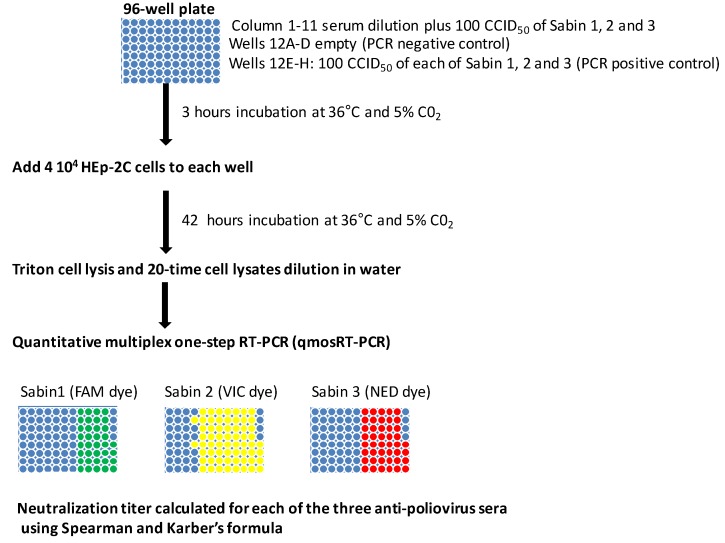
Multiplex PCR-based neutralization (MPBN) assay layout.

The proposed poliovirus MPBN assay is depicted in Figure 3. The assay is performed in 96-well plates and uses all three Sabin strains in mixture as challenge-virus. It is able to determine the titers of the three anti-poliovirus sera types in the same reaction.

### 3.2. Comparison of MPBN with Conventional Neutralization Assay

The determination of titers of trivalent human serum that has antibodies against all three poliovirus serotypes was performed by MPBN assay using different combinations of challenge Sabin strains and three PCR replicates for each combination of Sabin strains. The resulting titers for each combination of challenge viruses and the results obtained using the conventional neutralization assay are summarized in Table 2. Additionally, the titer determination of anti-poliovirus rat and rabbit sera by MPBN assay was performed using the three Sabin strains mixture as the virus challenge. The resultant titers were compared to the results of the conventional neutralization assay, as presented in Table 3.

The results demonstrate that the MPBN assay and the standard neutralization test produced similar results. The MPBN assay produced consistent and reproducible results (less than two-fold variation) even with different combination of challenge viruses and reduced the time to get results to 2–3 days instead of 10 days for the standard neutralization test. It produced neutralization titers simultaneously for sera of anti-poliovirus types 1, 2 and 3.

### 3.3. Correlation between Titration Results of the Conventional and MPBN Assays

**Figure 4:**
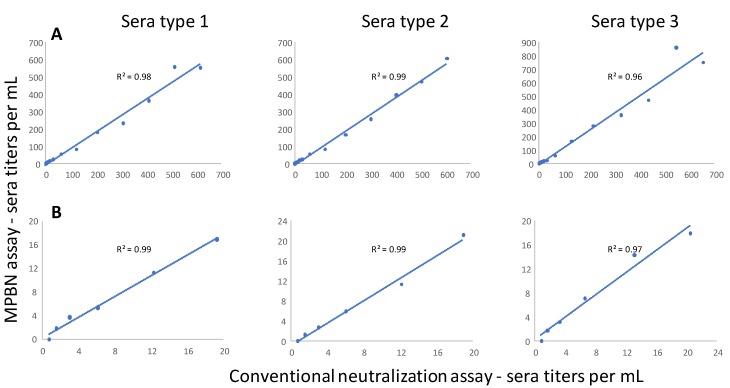
(**A**)**:** Evaluation of correlation between titration results of the conventional and MPBN assays. (**B**): Correlation of results of low-titer sera (from the data presented in A) analyzed with the conventional and MPBN assays.

We assessed the correlation between MPBN and conventional neutralization assays using anti-poliovirus trivalent serum (serum of rabbit immunized with mixture of the three poliovirus serotypes) with known titers previously determined with a conventional neutralization assay. The serum was serially diluted, and each dilution was subjected to an MPBN assay, as described above. The results of the MPBN assay were plotted against the known conventional titers of the corresponding dilutions for each serum type, as presented in Figure 4A. Excellent correlation was observed between the two assays for each of the three sera types, with R^2^ values of 0.98 for type 1 and 0.99 for type 2 and 0.96 for type 3. The estimated slopes and associated 95% confidence intervals are 0.94 (0.86 and 1.03), 0.96 (0.91 and 1.01) and 1.27 (1.10 and 1.43) for poliovirus types 1, 2 and 3, respectively, suggesting no proportional bias between the two methods.

For a more detailed analysis, the results for low-titer sera (from the data presented in Figure 4A) are presented separately in Figure 4B. These data show that the results of both assays correlate well with R^2^ values of 0.96 for serum type 1, 0.99 for type 2 and 0.97 for type 3. The results of the lower limit of titration (LLOT) analysis presented below are a part of this correlation study which showed that the MPBN assay accurately determined neutralization titers in low-titer sera.

### 3.4. Robustness of the MPBN Assay

We studied the influence of cell number variability on the MPBN assay results. To do this, we performed MPBN assays for the analysis of anti-poliovirus trivalent sera but added a varying number of cells into the wells (Table 4). The results showed that the MPBN assay generated similar results (≤two-fold difference) if the number of HEp-2C cells varied between 1 × 10^5^ and 2 × 10^4^. We also investigated the effects of delay in adding cells after three hours of incubation of the sera with poliovirus. Delays of 30 min to 1 h were studied and showed no effect on titration results (Table 5).

### 3.5. MPBN Specificity and Sensitivity to Determine the Titers of the Three Types of Anti-Poliovirus Sera

The previous characterization of the qmosRT-PCR method showed that the method is very sensitive, specific and has a very large linearity range for simultaneous identification and quantitation of all three Sabin strains [18]. In this work, the specificity of qmoRT-PCR to identify each Sabin strain in different combination of viruses was studied (Table 1).

The MPBN assay was found to be very specific in titration of anti-poliovirus antibodies to all three serotypes simultaneously (Table 2 and Table 3). Additionally, to determine the lower limit of titration (LLOT) of the MPBN assay, anti-poliovirus serum analyzed with conventional neutralization assay (having titers of 618 for type 1, 608 for type 2 and 657 for type 3) were diluted 50, 100, 200, 400 and 800 times and subjected to MPBN assay. Each serum dilution was analyzed by MPBN assay as a separate sample with the three types of Sabin strains together as challenge viruses in the same reaction. The results in Table 6 show that the LLOT measured with the MPBN assay was within 1.55–0.77 per mL for type 1, 1.52–0.76 per mL for type 2 and 1.64–0.82 per mL for type 3. The MPBN assay was able to titrate the three types of anti-poliovirus sera simultaneously in the same reaction with high sensitivity and specificity.

### 3.6. Consistency of MPBN Assay

Consistency of the MPBN assay was tested by titrating trivalent anti-poliovirus sera on three different days. Four independent tests were performed on day 1, four on day 2 and three on day 3. Results shown in Table 7 demonstrate that the MPBN assay generated consistent results for all three types of anti-poliovirus sera. We observed no significant differences in titers repeatedly obtained on the same day or on different days. Observed variations did not exceed two-fold difference. In short, the MBPN assay yielded consistent and reproducible titers.

## 4. Discussion

In 1988, WHO launched the Global Polio Eradication Initiative (GPEI) designed to eradicate poliomyelitis [22]. At that time, the annual global cases of paralytic poliomyelitis were estimated to be about 350,000 cases, with wild poliovirus (WPV) circulating in most countries. Extensive use of live, attenuated OPV in mass vaccination campaigns improved routine immunization programs and resulted in a gradual decrease of poliomyelitis cases. Presently, only Afghanistan and Pakistan are endemic with wild poliovirus type 1 [23], but outbreaks caused by virulent vaccine-derived polioviruses of all three types occur regularly. Wild poliovirus type 2 was last detected in 1999 and was declared eradicated in September 2015, while wild poliovirus type 3 has been eliminated since 2012 and declared eradicated in October 2019 [24]. Inactivated polio vaccine (IPV) is the only polio vaccine that has been given in the United States since the turn of the century. Many other countries still use OPV in their childhood immunization schedule, while others have gradually switched to IPV consistent with the WHO strategy to reduce the emergence of vaccine-derived polioviruses (VDPV) [25].

Epidemiological and seroprevalence surveillance are an essential part of GPEI [26]. It is crucial for monitoring the quality of vaccination campaigns and to identify regions that may require supplemental immunizations.

The level of population immunity required for poliovirus elimination must be about above 80% and depends on the level of hygiene [27]. A neutralizing-antibody titer higher than 1:8 is considered to be protective against paralytic poliomyelitis [28]. In laboratories, the neutralizing-antibody titer has been determined by a conventional poliovirus neutralization test using a susceptible cell culture system and infectious challenge virus [14].

The conventional poliovirus neutralization test is used to measure the ability of anti-poliovirus antibodies to inhibit virus cytopathic effects (CPE) in HEp-2c cells. Neutralizing antibody titers are expressed as the reciprocal of the highest serum dilution protecting half of the cell-wells from virus infection [29]. Virus titration and neutralization assays using CPE endpoint are simple but have some limitations. They are usually time-consuming (7–10 days for poliovirus), and their results are variable because of difficulties in evaluating CPE [16]. They cannot be used for viruses that do not cause CPE, and they cannot be used in multiplex format. Recently, for many viruses, these assays were modified to include a quantitative non-multiplex PCR for assessing virus replication [30,31,32,33,34]. Therefore, to overcome these limitations of the conventional assay, we developed the MPBN assay.

Similar to conventional neutralization assays, the MPBN assay tests serially diluted sera samples mixed with a predetermined amount of a mixture of the three Sabin strains and HEp-2c cells in replicate wells of 96-well plates, except that, instead of waiting ten days for the development of CPE, medium supernatant is discarded after 36–42 h of incubation, cells lysed and viral nucleic acids in cell lysates are quantified by qmosRT-PCR assay; results are used to determine sera titers expressed as the reciprocal of the highest serum dilution protecting cells from virus infection per mL. This way, serum titer is not determined based on proportionality to Ct (cycle threshold) values obtained by qmosRT-PCR, but rather, qmosRT-PCR is used as a readout to determine the presence or the absence of viral replication. A Ct of 40 is used as threshold; Ct less or equal 40 are considered positive for viral replication in the cells. Therefore, qmosRT-PCR results are expressed in yes or no format, allowing a simple Karber formula to be used similar to the conventional neutralization assay.

The MPBN assay proved to be very sensitive, accurately titrating an equivalent of anti-poliovirus sera of 1.55–0.77 per mL for type 1, 1.52–0.76 per mL for type 2 and 1.64–0.82 per mL for type 3 in mixtures (sera against the three poliovirus serotypes together). In addition, the MPBN method generated titers similar (within the margin of a single dilution) to those of conventional assays for all combinations of Sabin challenge strains.

We found excellent correlations between the conventional and MPBN assays for anti-poliovirus sera of all three serotypes with an R^2^ value of 0.98 for type 1, 0.99 for Sabin 2 and 0.96 for type 3. This correlation was observed for sera with both high and low titers. The LLOT was within the linear range, indicating that the assay can accurately titrate samples with low titers. The MPBN assay generated consistent and reproducible results for all three sera types and proved to be very robust. Even a one-hour delay in the addition of cells following incubation of the sera-viruses complex, or a five-fold variation in the number of cells, had no effect on neutralization titers (Table 4 and Table 5). This method reduced the time needed to titrate sera against polioviruses from the seven to ten days for conventional neutralization assay to only two to three days. Importantly, the MPBN assay accurately titrated the sera of all three poliovirus serotypes simultaneously in the same reaction-run.

The MPBN assay can be used to analyze seroprevalence of anti-poliovirus antibodies and seroconversion rates in populations. It can also be used for analysis of sera collected from clinical studies of a new generation of polio vaccines and for analysis of animal immunogenicity studies, such as the rat IPV potency assay. The ability to obtain results in just 2–3 days and its multiplex design can make MPBN attractive for time-critical and high-throughput applications.

## 5. Conclusions

The MPBN assay described in this communication offers a simple and rapid alternative to traditional neutralization assays to detect, identify and titrate either individual or combined sera against the three poliovirus serotypes. The MPBN method is well-suited to quantify anti-poliovirus sera in the many samples collected during clinical trials of new poliovirus vaccines and during routine clinical and seroprevalence studies of poliovirus surveillance programs. The MPBN assay can also be applied during manufacture of poliovirus vaccines for quality control by evaluating their potency and immunogenicity. This assay is suitable for automation, facilitating high-throughput applications, improving consistency of titrations and saving time and labor. The MPBN method can also be applied to titrate sera against other viruses, including those that produce no CPE.

## Figures and Tables

**Table 1:** qmosRT-PCR identification of Sabin strains in duplicates using different combinations of purified RNA and infected cell lysates.

Samples	Ct of Sabin 1	Ct of Sabin 2	Ct of Sabin 3
**Sabin 1 RNA**	15.55	UD	UD
	15.61	UD	UD
**Sabin 2 RNA**	UD	15.32	UD
	UD	15.12	UD
**Sabin 3 RNA**	UD	UD	15.72
	UD	UD	15.53
**Sabin 1 & 2 RNA**	17.72	15.24	UD
	17.75	15.23	UD
**Sabin 2 & 3 RNA**	UD	15.45	16.12
	UD	15.74	15.91
**Sabin 1 & 3 RNA**	16.90	UD	15.94
	16.49	UD	16.11
**Sabin 1, 2 & 3 RNA**	19.18	15.73	16.71
	19.46	15.94	16.94
Sabin 1—HEp-2C *	24.56	UD	UD
	24.00	UD	UD
Sabin 2—HEp-2C *	UD	25.27	UD
	UD	25.10	UD
Sabin 3—HEp-2C *	UD	UD	23.20
	UD	UD	23.89
Sabin 1, 2 & 3—HEp-2C *	27.40	27.45	23.65
	29.14	27.46	23.00
**NC**	UD	UD	UD
	UD	UD	UD

**Note**: *: lysates of infected HEp-2C cells, UD: undetermined, NC: negative control and Ct: quantitative PCR cycle threshold.

**Table 2:** Titration of human anti-poliovirus sera type 1, 2 and 3 with multiplex PCR-based neutralization (MPBN) and conventional neutralization assays.

Virus Challenge	PCR Repeats	Sera Titers		
		Type 1	Type 2	Type 3
**MPBN Assay**				
**Sabin 1, 2 and 3**	1	14,596	5793	7298
	2	14,596	5793	7298
	3	14,596	5793	5793
**Sabin 1 and 2**	1	9195	5793	
	2	9195	5793	
	3	9195	5793	
**Sabin 2 and 3**	1		5793	7298
	2		5793	7298
	3		5793	7298
**Sabin 1 and 3**	1	11,585		7298
	2	11,585		7298
	3	11,585		5793
**Sabin 1**	1	11,585		
	2	11,585		
	3	11,585		
**Sabin 2**	1		5793	
	2		5793	
	3		5793	
**Sabin 3**	1			5793
	2			5793
	3			5793
**Conventional Neutralization Assay**				
**Sabin 1, 2 and 3**	NA	11,585	5793	13,777
**Mean**		11,729	5793	7218
**STDEV**		1916	0	2106

**Note:** STDEV; Standard Deviation.

**Table 3:** Titration of rat and rabbit anti-poliovirus sera type 1, 2 and 3 with MPBN and conventional neutralization assays.

Rat Sera *	Assay	Virus Challenge	Serum Titer Per mL		
			Type 1	Type 2	Type 3
**29**	MPBN	Sabin 1, 2 & 3	912	114	228
	Conventional		724	91	181
**31**	MPBN	Sabin 1, 2 & 3	2	1448	23
	Conventional		2	1024	16
**32**	MPBN	Sabin 1, 2 & 3	16	128	32
	Conventional		11	128	32
**33**	MPBN	Sabin 1, 2 & 3	23	362	23
	Conventional		16	256	23
**36**	MPBN	Sabin 1, 2 & 3	128	91	1024
	Conventional		181	156	512
**37**	MPBN	Sabin 1, 2 & 3	0	362	57
	Conventional		0	256	23
**2**	MPBN	Sabin 1, 2 & 3	181	512	45
	Conventional		144	362	64
**3**	MPBN	Sabin 1, 2 & 3	118	724	362
	Conventional		181	724	512
**4**	MPBN	Sabin 1, 2 & 3	64	256	362
	Conventional		64	724	181
**5**	MPBN	Sabin 1, 2 & 3	0	512	23
	Conventional		0	575	23
**8**	MPBN	Sabin 1, 2 & 3	181	32	91
	Conventional		256	45	45
**Trivalent Rabbit Sera ˠ**	MPBN	Sabin 1, 2 & 3	554	604	750
	Conventional		618	608	657
**Monovalent Rabbit Sera ˠ**	MPBN	Sabin 1	1086		
		Sabin 2		1183	
		Sabin 3			2057
	Conventional		1086	1435	1573

**Note**: *: sera from rats immunized with inactivated poliovirus vaccine, ˠ: sera from rabbits immunized with the three wild types of poliovirus and Sabin 1, 2 & 3: the mixture of the three Sabin strains.

**Table 4:** Impact of HEp-2C cell number variability on anti-poliovirus sera titers generated by MPBN assay.

Serum Type	Hep-2C Cells Number				Mean	STDEV
	1.0 × 10^5^	8.0 × 10^4^	4.0 × 10^4^	2.0 × 10^4^		
**1**	813	575	645	724	689	103
**2**	724	645	813	1290	868	290
**3**	645	1024	1024	645	835	219

**Table 5:** Impact of the delay in adding cells after the three hours of incubation of the sera with poliovirus on the MPBN assay results.

Serum Type	Incubation Time (Hours)			Mean	STDEV
	3:00 *	3:30	4:00		
**1**	**645**	813	767	742	87
**2**	724	724	1024	824	173
**3**	813	832	1085	910	152

**Note**: *: three hours is the standard incubation time for the conventional neutralization and MPBN assays.

**Table 6:** Determination of the low limit of titration (LLOT) of anti-poliovirus sera types 1, 2 and 3 by MPBN assay.

Serum Type	Assay	Serum Titer Per mL				
**1**	**Conventional**	12.36	6.18	3.09	1.55	0.77
	**MPBN**	14.25	5.34	3.77	1.80	0.00
**2**	**Conventional**	12.16	6.08	3.04	1.52	0.76
	**MPBN**	13.45	5.99	2.83	1.40	0.00
**3**	**Conventional**	13.14	6.57	3.29	1.64	0.82
	**MPBN**	17.90	7.13	3.17	1.80	0.00

**Table 7:** Results of consistency evaluation of MPBN assay using anti-poliovirus trivalent serum.

Day	Run	Titer of Anti-Poliovirus Serum Per mL		
		Type 1	Type 2	Type 3
**1**	1	575	609	861
	2	431	456	912
	3	483	483	724
	4	362	575	861
**2**	1	542	512	483
	2	362	542	542
	3	456	383	542
	4	456	431	542
**3**	1	609	496	724
	2	542	599	683
	3	542	679	575
**Mean**		487	524	677
**STDEV**		82	87	152

**Note:** STDEV; Standard Deviation.
